# Using a Prediction Model to Manage Cyber Security Threats

**DOI:** 10.1155/2015/703713

**Published:** 2015-05-03

**Authors:** Venkatesh Jaganathan, Priyesh Cherurveettil, Premapriya Muthu Sivashanmugam

**Affiliations:** Department of Management Studies, Anna University Regional Centre Coimbatore, Jothipuram Post, Coimbatore, Tamilnadu 641 047, India

## Abstract

Cyber-attacks are an important issue faced by all organizations. Securing information systems is critical. Organizations should be able to understand the ecosystem and predict attacks. Predicting attacks quantitatively should be part of risk management. The cost impact due to worms, viruses, or other malicious software is significant. This paper proposes a mathematical model to predict the impact of an attack based on significant factors that influence cyber security. This model also considers the environmental information required. It is generalized and can be customized to the needs of the individual organization.

## 1. Introduction

The digital assets of an organization are prone to attack any time. With threats gathering new dimensions, organizations should be able to objectively evaluate the risks of existing and new software applications. Based on this risk evaluation, sufficient resources can be allocated to mitigate cyber security risks. Quantitatively predicting proneness to attack can help organizations counter attack occurrences. The Common Vulnerability Scoring System (CVSS) is a standard framework used by many organizations. It communicates the characteristics and impacts of IT vulnerabilities. This framework has three groups, namely, Base, Temporal, and Environmental. The base group highlights the qualities of vulnerability that are unchanged over time and user. The temporal group covers the characteristics of vulnerability over time and the environmental group highlights the specific user environment. The CVSS helps establish a common language in the IT community. This paper proposes a mathematical model for predicting the impact of an attack based on the significant factors that influence cyber security. These factors are arrived at by considering several historical data points and mathematically verifying their significance to the impact and characteristics of attacks.

## 2. Related Work

Sheyner et al., Wang et al., and Kuhl et al. [1–3] highlighted that security analysis is based on attack graph generation and simulation. The focus of their work was on attack generation tools. This paper proposes establishing the quantitative relationship between the attack impact and the attack parameters. Tittel [4] explains about Unified Threat Management and why it is important to address it in his paper. Wu et al. [5] established a prediction model based on integrating environmental factors and attack graphs in a Bayesian network. They found that environmental information is important for accurate safety evaluations. This paper proposes mathematically proving that environmental information influences the characteristics and impacts of an attack. Anusha et al. [6] studied various models like unimodel and multimodel for enhanced security. They discussed about authentication at the beginning of the exam and the user system checks. Axelrad et al. [7] introduced a Bayesian network model for the motivation and psychology of the malicious insider. Khan and Hussain [8] established relationships between attack probability and vulnerability. However, the collective influence of attack environment factors on the attack was not revealed. Moore et al. [9] described a modeling and simulation foundation, based on the system dynamics methodology to test the efficacy of insider threat detection controls. The paper discusses risk management and early detection of risks based on insider threat.

The three impact metrics in the CVSS measure how vulnerability is assessed and how it impacts on an IT asset. These three metrics are Access Vector, Access Complexity, and Authentication. It is also important to understand how vulnerability affects the integrity, confidentiality, and availability of these parameters. Cyber security metrics can be broadly classified into two based on the source of measurement. Several measurements are possible from the malicious user/group end. Some measurements are also possible from the host/victim end. From the side of a malicious user [10], the Intensity, Stealth and Time of attack are possible measurements. Further, the technical and cyber knowledge of personnel are relative measurements from the malicious user/group end. Measurements that are possible from the host end include the Vulnerabilities present in the tool, which are detectable through tools such as Nessus and X-Force, the Traffic to a particular application over a period of time, the Power of the protection tools installed at the target system, and the Value of the assets present in the network. From these two classifications, to build a prediction model, the measurement taken from the host end is used, whereas none of the malicious user/group end measurements is considered. With the increasing dimensions of attack nature, it is not possible to accurately calculate these measurements. Moreover, such measurements from the malicious user end are of little use if the attack possibilities need to be controlled by organizations hosting the target applications.

## 3. Prediction Model

Prediction models can be developed to predict different project outcomes and interim outcomes by using statistical techniques. A process performance model adopts the concepts of probability. This can also be explored further by building simulations. Output can be studied as a range. Depending on the predictions, midcourse corrections can be recommended. The model can be simulated to predict final outcomes based on the corrections suggested. It is thus a proactive model that helps the technical analyst to analyze the data and predict outcomes. Analysts can change the data and perform what-if analyses. They can then record these instances and decide on the best option. The model helps analysts decide which lever to adjust to meet the final project goal.

In the current system, the focus of fixing vulnerabilities is not based on the potential impact of the vulnerability. Further, more than adequate importance is given to fixing all the vulnerabilities or too little importance due to time and cost constraints. A proactive risk assessment prior to the release of the IT application is not available. The impact of any vulnerability is identified by using the CVSS calculator, only after analyzing how the attack took place.

In the proposed model, which was also piloted in a sample project organization, the potential impact of the vulnerability is predicted well before the IT application is released for usage. With this impact information, adequate cost and resources can be allocated to resolving vulnerabilities, thereby reducing the impact.

The multiple regression method is chosen to predict the impact of attacks in this proposal. Multiple regressions have certain underlying assumptions such as linearity, the nonexistence of multicollinearity, homoscedasticity, and normality. Each of these assumptions is validated for our attempt to establish the relationship between the impact of attack and the influencing factors. Based on the research above, factors that can influence the impact of an attack were identified, namely, Level of security protection on the target system, Usage or traffic in the identified network, Vulnerabilities present in the target system, and Value of assets present in the network. Oluwatosin and Samson [11] highlighted the challenges of existing computer applications that need to be considered from a security perspective.

Shar et al. [12] predicted vulnerabilities with features related to dataflow. Khan and Hussain [8] stated that it is safe to assume that all these factors have individual linear relationships with the probability of attack. A similar relationship is seen from the scatterplot of the impact of an attack and the environmental factors considered. This satisfies the first condition for using the multiple regression technique for our prediction model.

Multicollinearity and homoscedasticity are verified through the variance inflation factor (VIF). Normality is tested by performing the Anderson–Darling Test. In addition, a hypothesis test is run individually for each of the attack factors to ensure that the probability that the factor does not influence the independent factor is kept to a maximum of only 0.05. The coefficient of correlation (*R* ^2^) and adjusted coefficient of correlation (Adj. *R* ^2^) are maintained close to each other. This is sufficient to prove that among the many factors that can cause an attack, the selected factors predict the impact of an attack with the best possible accuracy. That is, the number of factors, beyond a certain point, becomes immaterial.

The operational definition of cyber security metrics considered is mentioned below.

1. *Y* is the Overall CVSS Score, the dependent factor. The CVSS [13, 14] is predicted based on the environment and system characteristics of the target application.
2. *X*1 is the number of vulnerabilities, namely, the total number of vulnerabilities detected by the static and dynamic vulnerability detection tools for the target application. The tools installed and run against the target application can identify several vulnerabilities based on algorithms such as but not limited to improved tainted algorithms or penetration testing. In a given application, the vulnerabilities reported by the tools can be broadly classified into 23 categories: API Abuse, Authentication Vulnerability, Authorization Vulnerability, Availability Vulnerability, Code Permission Vulnerability, Code Quality Vulnerability [15], Configuration Vulnerability, Cryptographic Vulnerability, Encoding Vulnerability, Environmental Vulnerability, Error Handling Vulnerability, General Logic Error Vulnerability, Input Validation Vulnerability, Logging and Auditing Vulnerability, Password Management Vulnerability, Path Vulnerability, Protocol Errors, Range and Type Error Vulnerability, Sensitive Data Protection Vulnerability [16], Session Management Vulnerability, Synchronization and Timing Vulnerability, Unsafe Mobile Code and Use of Dangerous API [17].
3. *X*2 is the Average Input Network Traffic recorded for the application during the week of the attack in KBPS.

Table 1 highlights the data points for each metric during all instances of an attack. In total, 25 such attack history data from a project are shown in Table 1. The data points from the CVSS calculator were recorded for every attack encountered. Output from the vulnerability tool was recorded for the target application. For the specified week range of the attack, network traffic was also recorded. The data from these three sources were tabulated every time an attack was encountered, as shown in Table 1. We also ensured that respective data points were taken from the same sample. The sample definitions were defined by the technical analyst. In this regression model, CVSS score (*Y*) was predicted by using the two *X* variables, vulnerability and network traffic.

**Figure 1:**
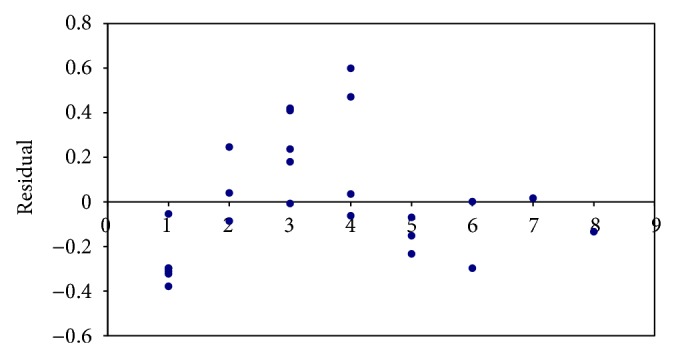
Residual plot.

**Figure 2:**
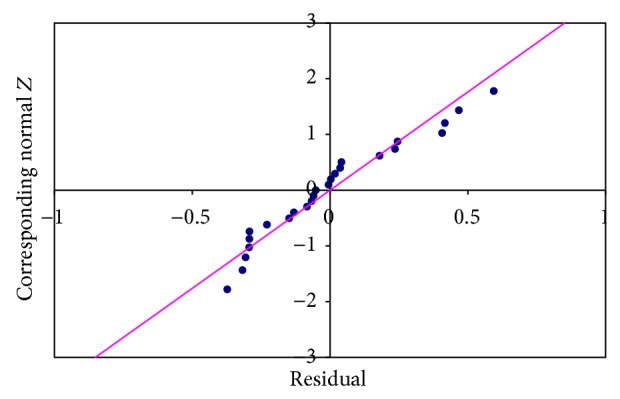
Normal probability plot.

The null hypothesis considered is that *X*1 and *X*2 have no influence over *Y*. In other words, vulnerability and network traffic have no influence over CVSS score. No mirror pattern can be found in the residual plot in Figure 1 and hence no heteroscedasticity is found. The normal probability plot shown in Figure 2 is approximately linear. From the figure, it is clear that the normality assumption for the errors has not been violated. With regard to the *P* value, since it is 0.02 (<0.05), the null hypothesis is not valid, which means the variables selected have an impact on CVSS score.

As shown in Table 2, vulnerability has a positive influence on CVSS score. As vulnerability increases, CVSS score increases and hence the impact on IT assets is high. The influence of network traffic on CVSS is positive. This means that when network traffic is high, the impact of vulnerabilities is high and CVSS score is high. Thus, CVSS score is impacted positively both by vulnerability and by network traffic:

(1) Predicted  CVSS  Score =−0.2893+0.07174∗Number  of  vulnerabilities    on⁡  the  IT  application  reported  by  tools  +0.0025∗Proposed  average  input  network  traffic    for  the  application  for  a  week  measured  in  KBPS.

As shown in Table 3, *b* is the coefficient that gives the least squares estimates, while *s*(*b*) gives the standard errors of the least squares estimates for the *x* variables and *t* gives the computed *t*-statistic. This is the coefficient divided by the standard error. The *P* value gives the *P* value for the hypothesis test. The VIF quantifies the severity of multicollinearity in an ordinary least squares regression analysis. The VIF for the given data is 6.41.

As shown in Table 4, SS is the sum of squares due to the regression. This measure of total variation in *Y* can be explained by the regression with the *X* variable. Df is the degrees of freedom. MS is the mean square, which is a measure of the sum of squares divided by the degrees of freedom. Mean square regression (MSR) and mean square error (MSE) are the two variables that define *F*: *F* = MSR/MSE. The *F*-statistic is used to test whether the *Y* and *X* variables are related.

For the given data, MSR is 48 and MSE is 0.08. The *F*-statistic determines that the *P* value is zero. This confirms the existence of a linear relationship between CVSS and the two variables, network traffic and vulnerabilities. *R* ^2^ provides information about the goodness of fit of a model. In the regression equation, the *R* ^2^ coefficient of determination determines how well the regression line approximates the real data points. Adjusted *R* ^2^ is a modified version that adjusts the number of predictors in the model. For the given data, *R* ^2^ is 0.9819 and adjusted *R* ^2^ is 0.9803.

The data prove that overall CVSS score is influenced by vulnerabilities in the network and network traffic. The infrastructure team in the organization shares the baseline data for these variables on a regular basis with the quality team. For each network process, based on the network type and applications hosted, a logical grouping can be considered and organization values can be baselined. Technical analysts can then refer to these baseline organizational data when they start the network design process. As part of the process, they can also use these reference values to determine the upper and lower specification limits. These values will be available for each of the subprocess parameters. The technical analyst can then determine and analyze which vulnerabilities need to be controlled and select threshold values based on that.

Based on the selected threshold values, what-if analysis is performed. Going by the different scenarios, vulnerability and network traffic values are assumed and provided as inputs to the model. The predicted outcome is then compared with the thresholds. It is important to note that while changing the parameters, technical analysts should understand the practical implications of the project. It is not only about the mathematical model, but about how it can be put into practice. For example, if CVSS score is high, how can it be reduced? How can vulnerabilities be reduced during design? Cost implications need to be considered. Then, the technical analyst has to look at the environmental constraints. As the prediction model considers the key influencing factors to predict the CVSS, the influencing factor values might affect the project schedule and project cost, which need to be analyzed as well. These forecasts serve as alerts that it should take action to mitigate the threat of cyber-attacks.

Predicting CVSS scores helps prioritize vulnerabilities and remediate those with high risks. CVSS scores are shared by software application vendors with their customers. This helps customers understand the severity of vulnerabilities and allows them to effectively manage their risks. Vulnerability bulletins are shared by few organizations. These bulletins share the date of attack, systems affected, and patches performed. Thus, the CVSS prediction model is vital and should be used extensively. Technical analysts should be comfortable using the prediction model extensively. For every scenario, the analyst should document the assumptions and associated risks. A detailed attack prevention plan should be in place. At every step, the attack, its type, cause, and preventive action should be documented. Different root-cause analysis techniques such as 5-why can be used to pinpoint the root cause. After identifying the root cause, the next steps in terms of corrective and preventive actions should also be thought through. Technical experts should review these plans so that they can bring in their experience and highlight any improvements.

Prediction models should not be a one-time activity. Technical analysts should use the model on an ongoing basis and also suggest shortcomings. Based on the scores, decisions need to be taken considering impact on cost and security. Prediction models are statistical and simulative in nature. These models should help simulating scenarios as well as determining outcomes. They can also model different variation factors and help the analyst with the predicted range or the variation of its outcomes.

## 4. Conclusion

Cyber-attack is an attempt to exploit computer systems and networks. Cyber-attacks use malicious codes to alter algorithms, logic, or data. Securing information systems is thus critical. Multiple countermeasures need to be built. The CVSS is an industry framework that helps quantify the vulnerability impact. This paper demonstrated a mathematical model to predict the impact of an attack based on significant factors that influence cyber security. Vulnerability and network traffic were selected as the influencing factors to predict CVSS score. Based on the score, the technical analyst can analyze the impact and take necessary preventive actions. This model also considers the environmental information required. It is thus generalized and can be customized to the needs of the individual organization.

## Figures and Tables

**Table 1:** Project data points.

*Y*	*X*1	*X*2
CVSS Score	Vulnerability	Network Traffic
2.1	20	324
5.3	53	623
1.0	15	235
8.0	85	932
2.9	28	438
3.0	25	498
3.8	38	391
1.0	18	132
1.2	16	177
5.9	63	823
4.3	39	579
2.8	30	455
1.1	14	231
4.2	35	725
5.4	51	740
1.9	21	345
2.0	25	432
4.1	37	467
6.2	58	845
1.1	15	111
2.3	22	191
1.2	16	182
2.8	30	292
6.9	68	952
4.8	55	600

**Table 2:** Regression equation.

Intercept	Vulnerability	Network Traffic
−0.2983	0.07174	0.0025

**Table 3:** Multiple regression results.

	Intercept	Vulnerability	Network Traffic
*b*	−0.296	0.0706	0.002
*s*(*b*)	0.121	0.007	0.0005
*t*	−2.442	9.359	4.545
*P*	0.0231	0.0000	0.0002

**Table 4:** ANOVA table.

Source	SS	Df	MS	*F*	*P*
Regression.	96.22	2	48.15	597	0.000
Error	1.77	22	0.080		

Total	98	24			
